# Wisdom from the past

**Published:** 1971-07

**Authors:** 


### Wisdom From The Past

(Extracts from This Journal 1896 and 1921)

### SEVENTY FIVE YEARS AGO

### NOTES ON PREPARATIONS FOR THE SICK

**L.C.C. Meat Juice** — The Liquor Carnis Company, Aston Clinton, Bucks. This very dark-coloured, opaque, viscid fluid is described by its vendors as a "liquid asset", whilst cooked foods, essences, and extracts are, as it were, "bills to be discounted." It certainly is a very concentrated fluid, as when diluted to four times its bulk its specific gravity is as high as 1060. It precipitates freely with picric acid, the albuminometer tube showing a percentage of .4 per cent. in the diluted fluid, making the very high amount of 1.6 per cent. of albumen in the undiluted juice. It contains a quantity of granular sediment with fragments of cells and a few crystals, but we did not identify any fragments of striped muscle or unbroken red blood-cells. The sprectroscope does not show the ordinary haemoglobin bands. The makers say that the "juice consists of the uncooked juices of British beef, highly concentrated, obtained by hydraulic pressure, excluding the indigestible fibre, and retaining only the nutricious portions of the meat in a form that admits of direct absorption." Our examination of the fluid tends to support the above statement: it is the most concentrated fluid we have met with. It has a beefy aroma and flavour, and it may be taken diluted as a cold drink, or to supply the deficient nourishing qualities of a cup or ordinary home-made hot beef tea, the flavour of which is thereby improved.

**Gaskin's Cocoa; "Viking" Condensed Unsweetened Milk** — Anderson & Coltman, London. The chemical composition of this Cocoa is shewn by the following analysis (Granville H. Sharpe, F.C.S.) :

| | Per cent. | | Per cent. |
|---|---|---|---|
| Albuminous Matter | 23.75 | Water | 4.45 |
| Cocoa Butter | 31.26 | Ash | 4.43 |
| Theobromin | 1.05 | Other Nutritive Matters | 35.06 |

It is manufactured in British Guiana; is pure, retaining the flavour and aroma of the cocoa bean; it is very nourishing, as shewn by its composition; is easily digested; and one invalid, whom we know and who likes few things, says that this cocoa is very nice.

The Milk, from cows grazing in the Norwegian Highlands, unskimmed and condensed without the addition of sugar or any other substance, looks like cream, and when mixed with two or three parts of water it not only closely resembles but also has the chemical composition of fresh milk. Being sterilised, it will keep for any length of time, and may be kept in readiness so as to be at any time available for children and invalids who require sterilised milk of excellent quality. It differs from the ordinary brands in that it does not contain any admixture of cane sugar or any other preservative. For cooking and all other purposes it may be looked upon practically as concentrated sterilised milk.

**Australian Brandy** — Joshua Brothers, London. — It is only natural that the steady development of the Victorian vineyards should lead to the introduction of an Australian genuine grape brandy at a time when the shipments from the Cognac district have so much fallen off. The sample forwarded to us appears to be pure, and certainly its flavour is excellent. Its quality, its price, and the innate love which the English people have for things which are not foreign should enable this fluid to compete successfully with Scotch and Irish whisky.

(Vol. 14, pp. 279-80; 1896).

### FIFTY YEARS AGO

### From the Long Fox Lecture of 1921 on The Repair of Bone Injuries by Ernest W. Hey Groves, M.S., F.R.C.S.

"Five thousand years ago a young man in Egypt suffered a fracture of his radius, and was treated in the most approved fashion of the time by having his arm splinted by the central rib of a palm leaf. Shortly after this he met his death, and his bone, still supported by the primitive splint, is now to be seen in the Royal College of Surgeons. Eight hundred years ago in a village of Wessex a woman who had fractured her humerus had it bandaged up and then surrounded by two plates of thin copper. The remains can still be seen in the Museum at Reading.

"This is a fair representation of the treatment of bone injuries up till twenty-five years ago, when two great factors were introduced which greatly changed our conception of the actual treatment of bone repair. One, the introduction of the X-rays; the other, the general adoption of Industrial Insurance. Both these factors served to show that bone repair was often unsatisfactory, and led to a very great crippling and loss of function.

"During the last generation advance has been along four roads. In the first, the treatment of fractures in which there has been little or no displacement has been by massage and early movement rather than splinting, and the improvement produced by this practice, chiefly associated with the name of Lucas-Championniere, has not merely been due to the special type of massage and early movement, but also to the negative factor of the absence of immobilisation. The second road along which knowledge developed is that for gross displacements direct open operative treatment has been devised. Thirdly, that for the great majority of fractures with serious displacement traction systems, by which the end of the bones are held apart from one another, have been introduced. Lastly, for those cases which either by injury or by action of disease present a loss of substance the treatment by bone grafting has been devised and brought to a state of practical utility."

(Vol. 38, pp. 142-143; 1921).

